# LECT2 Protects Nile Tilapia (*Oreochromis niloticus*) Against *Streptococcus agalatiae* Infection

**DOI:** 10.3389/fimmu.2021.667781

**Published:** 2021-05-20

**Authors:** Qi Li, Zhiqiang Zhang, Weiqi Fan, Yongxiong Huang, Jinzhong Niu, Guoling Luo, Xinchao Liu, Yu Huang, Jichang Jian

**Affiliations:** ^1^ College of Fishery, Guangdong Ocean University, Guangdong Provincial Key Laboratory of Pathogenic Biology and Epidemiology for Aquatic Economic Animal, Key Laboratory of Control for Disease of Aquatic Animals of Guangdong Higher Education Institutes, Southern Marine Science and Engineering Guangdong Laboratory, Zhanjiang, China; ^2^ Laboratory for Marine Biology and Biotechnology, Qingdao National Laboratory for Marine Science and Technology, Qingdao, China; ^3^ Guangdong Provincial Engineering Research Center for Aquatic Animal Health Assessment, Shenzhen, China

**Keywords:** LECT2, Nile tilapia, *Streptococcus agalactiae*, immune response, CLR

## Abstract

Leukocyte cell-derived chemotaxin 2 (LECT2) is a multifunctional cytokine that especially plays an important role in innate immune. However, the roles of LECT2 in the immune response of the economically important fish Nile tilapia (*Oreochromis niloticus*) against bacterial infection remains unclear. In this study, a *lect2* gene from Nile tilapia (*On-lect2*) was identified, and its roles in the fish’s immune response against bacterial infection were determined and characterised. *On-lect2* contains an open reading frame of 456 bp that encodes a peptide of 151 amino acids, as well as the conservative peptidase M23 domain. On-LECT2 is 62%–84% identical to other fish species and about 50% identical to mammals. The highest transcriptional level of *On-lect2* was detected in the liver, whereas the lowest levels were detected in the other tissues. Moreover, the On-LECT2 protein is located mainly in the brain and head kidney. The transcriptional levels of *On-lect2* substantially increased in the head kidney, brain, liver and spleen after *Streptococcus agalactiae* infection. Knockdown *On-lect2* led to higher mortality due to liver necrosis or haemorrhage and splenomegaly. *In vitro* analysis indicated that the recombinant protein of On-LECT2 improved phagocytic activity of head kidney-derived macrophages. *In vivo* challenge experiments revealed several functions of On-LECT2 in the immune response of Nile tilapia against bacterial infection, including promotion of inflammation, reduction of tissue damages and improvement of survival rate.

## Introduction

Leukocyte cell-derived chemotaxin 2 (LECT2) is a member of the peptidase M23 family, first isolated from the human T cell line SKW-3 and initially identified as a chemotaxin of neutrophils that plays a role in the regulation of neutrophil chemotaxis *in vitro* (1). Further studies established that the function of LECT2 is not just limited to chemotaxis (2). LECT2 is also involved in liver regeneration (3–6), immune response (7, 8), bone growth (9), and liver tumorigenesis (10, 11). Moreover, LECT2 can be regarded as a marker of various diseases in clinical applications (11–15). Although the peptidase M23 domain is conserved, LECT2 has not been found to possess enzymatic activity like other metalloendopeptidases (16). However, the conserved HxGxDx motif of LECT2 plays crucial roles in the pathogenesis of hepatocellular carcinoma as a tumour suppressor (17).

LECT2 has been identified from various organisms, ranging from fish to humans (18–21), in which they appear to be highly evolutionarily conserved (2, 22). Mammals, cyprinid fish and catfish have six conserved cysteine residues, whereas most teleost fish species lack the second and third cysteine residues (22, 23). In humans, *lect2* is located on chromosome 5 at position q31.1, close to several immune-related genes, and composed of four exons and three introns (21). *lect2* mRNA is highly expressed in the liver while lowly expressed in other tissues. By contrast, the LECT2 protein is widely expressed in various tissues, implying that most of the protein is immediately secreted after synthesised (24, 25). However, the regulation of the immune response of fish by LECT2 has not been studied as much as in mammals, a gap in research that restricts our understanding of the functions of this protein in teleosts.

Nile tilapia (*Oreochromis niloticus*) is an internationally farmed trade species recommended by the United Nations Food and Agriculture Organisation and an economically important fish in over 100 countries and regions, especially in China (26, 27). In recent years, outbreaks of bacterial diseases have led to leading huge economic losses and have threatened the development of tilapia culture (28). Since 2009, more than 90% of the clinical bacterial isolates from infected tilapia have been *S. agalactiae* (29). The most common symptoms caused by *S. agalactiae* include septicaemia, exophthalmia, ascites, spleen haemorrhage, in turn, lead to mortality (30). In this study, *O. niloticus* LECT2 was identified. Its roles in maintaining normal liver functions and regulating immune response against *S. agalactiae* infection were also investigated. These data may enhance our understanding of LECT2 functions in fish against bacterial infection.

## Materials and Methods

### Fish Preparation, Challenge and Sample Collection

All Nile tilapia (50 ± 5 g) individuals used in this work were acquired and fed in the same manner as we did in a previous study (31). The fish were randomly selected for subsequent experiment. All experiments were conducted according to the principles and procedures of Guangdong Province laboratory animal management regulations.

Three healthy fish were first narcotized with MS-222 (Sigma, Darmstadt, Germany) and then the tissue distribution of *On-lect2* in healthy tilapia was investigated by collecting tissues from brain, gills, heart, head kidney, intestine, liver, muscle, skin, spleen and thymus. Total RNA was immediately extracted.

*Streptococcus agalactiae* (ZQ0910) was isolated from Nile tilapia and kept in our laboratory (32). *S. agalactiae* was cultured in fresh brain heart infusion (BHI) liquid medium overnight, collected by centrifugation, washed three times in phosphate-buffered saline (PBS). Afterward, 100 µL of *S. agalactiae* with a final concentration of 5×10^7^ CFU/mL was injected into 30 fish. At five time points (0, 6, 12, 24 and 48 h) after injection, three fish were collected as mentioned above and then the brain, head kidney, liver and spleen tissues were collected. Total RNA was immediately extracted.

### RNA Isolation and cDNA Synthesis

Total RNA from Nile tilapia were isolated using RNAiso Plus (TaKaRa, Dalian, China). The RNA was then reverse-transcripted using PrimeScript™ RT reagent kit with gDNA Eraser (TaKaRa, Dalian, China) and diluted with distilled water at a ratio of 1:50 for subsequent experiments following the manufacturer’s instructions.

### Cloning and Sequence Analysis of *On-lect2*

The predicted gene sequence of *On-lect2* was obtained from NCBI (https://www.ncbi.nlm.nih.gov/nuccore/XM_003449406.5) and designed the partial sequence primers (On-lect2-S and On-lect2-A). Then the first strand cDNA of head kidney tissue was used as polymerase chain reaction (PCR) template to amplify *On-lect2* fragment and sequenced it. Using the sequenced sequence, the primers for amplifying the 5’ terminal sequence (lect2-5’ RACE and lect2-5’ RACE nested) and the 3’ terminal sequence (lect2-3’ RACE and lect2-3’ RACE nested) of *On-lect2* were designed respectively. For the 5’ and 3’ terminal sequence, the cloning method was referred to SMARTer RACE 5’/3’ Kit (Code No. 634858, Clontech, Mountain View, USA). Finally, the partial sequences acquired through gene cloning, 3’RACE and 5’RACE were assembled using contigExpress application software. All the primers used in this study were designed with the NCBI Primer designing tool (https://www.ncbi.nlm.nih.gov/tools/primer-blast/, **Table S1**). The potential open reading frame (ORF) of *On-lect2* was identified using the NCBI ORF finder (https://www.ncbi.nlm.nih.gov/orffinder/). Gene structure was plotted with Exon–Intron Graphic Maker (http://www.wormweb.org/exonintron). Molecular weight, theoretical p*I* and amino acid composition were predicted using the ProtParam tool (https://web.expasy.org/protparam/). The potential signal peptide was predicted with SignalP (http://www.cbs.dtu.dk/services/SignalP/). The potential transmembrane domain was predicted with TMHMM Server v. 2.0 (http://www.cbs.dtu.dk/services/TMHMM/). Multiple sequence alignments of LECT2 proteins among other species were conducted using DNAMAN software (version 7.0). Similarity among the determined amino acid sequences was analysed by UniProt (https://www.uniprot.org/). A neighbour-joining (NJ) phylogenetic tree was constructed with MEGA software (version 6.0) with 1,000 bootstrap replications.

### Quantitative Real-Time PCR (qRT-PCR)

The tissue distribution and relative expression of *On-lect2* in healthy tilapia at the transcriptional level was assessed *via* qRT-PCR. qRT-PCR was performed with TB Green^®^ Premix Ex *Taq™* II (Tli RNaseH Plus) (TaKaRa, Dalian, China) and QuantStudio 6 and 7 Flex Real-Time PCR Systems (Thermo Fisher Scientific, Waltham, USA) following the manufacturers’ instructions. A 187 bp fragment of *On-lect2* was amplified, and *On-β-actin* was used as a reference gene. The primers are listed in **Table S1**. Relative levels of *On-lect2* were calculated *via* the 2^-ΔΔCt^ method (33). These reactions were performed with three sample replicates and three technical replicates.

### Western Blot

The tissue distribution and relative expression of On-LECT2 in healthy tilapia at the protein level was assessed *via* Western blotting. Total protein from muscle, skin, brain, head kidney, liver and spleen tissues were isolated using a protein extraction kit (Solarbio, Beijing, China). Each sample containing 20 μg of total protein was loaded on 12% SDS-PAGE and transferred to a PVDF membrane (Millipore). The samples were blocked with QuickBlock™ Blocking Buffer for Western Blot (Beyotime, Shanghai, China) overnight at 4°C and then incubated with anti-LECT2 (PAF541Mu01, Cloud-Clone Corp, Wuhan, China) and anti-β-actin (AF5003, Beyotime, Shanghai, China) as primary antibodies. The solutions were diluted at a ratio of 1:1000 in QuickBlock™ Primary Antibody Dilution Buffer for Western Blot (Beyotime, Shanghai, China) for 2 h at room temperature. Afterwards, the membranes were washed three times in TBST and incubated with HRP-labelled goat anti-rabbit IgG (H+L) (Beyotime, Shanghai, China) at room temperature for 1 h. Antigen–antibody complexes were detected *via* the enhanced chemiluminescence method (P0018S, Beyotime, Shanghai, China).

### Histology and Immunohistochemistry

Brain, head kidney, liver and spleen tissues were collected from healthy or stimulated tilapia (challenged by *S. agalactiae* as mentioned above and RNAi as described below) and fixed in Dietrich’s fixative for over 20 h. The samples were dehydrated through a graded alcohol series (70%, 85%, 95% and 100%), cleared in xylene and then embedded in paraffin wax.

Serial sections 8 μm in thickness for histological analysis were stained with a haematoxylin and eosin staining kit (Beyotime, Shanghai, China) following the manufacturer’s protocols. The sections were observed and photographed using a Nikon DS-Ri2 microscope (Nikon, Tokyo, Japan).

Sections 5 μm in thickness for immunohistochemistry were rehydrated with PBT (PBS + 0.1% Tween-20). Endogenous peroxidase was inactivated with 3% H_2_O_2_, incubated with EDTA antigen retrieval solution (Beyotime, Shanghai, China) at 95°C to retrieve antigens and then incubated in PBT containing 3% bovine serum albumin (BSA). Subsequently, the primary antibody (anti-LECT2: PAF541Mu01, solutions were diluted at a ratio of 1:100 in 3% BSA; Cloud-Clone Corp, Wuhan, China) was added and incubated overnight at 4°C. Afterwards, the samples were washed five times in PBT and incubated with HRP-labelled goat anti-rabbit IgG (H+L) (Beyotime, Shanghai, China) at room temperature for 2 h. The samples were then washed three times in PBT, and antigen–antibody complexes were detected with DAB horseradish peroxidase colour development kit (Beyotime, Shanghai, China). Finally, the samples were stained with haematoxylin (Beyotime, Shanghai, China). Negative controls were performed by replacing the primary antibody with a preimmune serum to check antibody specificity. The samples were observed and photographed as mentioned above.

### RNAi Assay

Specific primers (**Table S1**) with the incomplete restriction sites *Bam* HI and *Hind* III were annealed with 50 µM of the final concentration to generate double-stranded oligo (ds oligo). Annealing was performed at 95°C for 5 min and then allowed to cool to room temperature for about 20 min. The RNAi plasmid pGenesil-1 (BT Lab, Wuhan, China) was digested with *Bam* HI and *Hind* III, ligated with ds oligo and transformed into *Escherichia coli* DH5α (TransGen, Beijing, China). The positive clone was verified *via* PCR and DNA sequencing. The RNAi plasmids pGenesil-1-shRNA-199/209/257/372 and the negative control plasmid pGenesil-1-shRNA-Control were extracted using E.Z.N.A.^®^ Endo-free plasmid Midi kit (Omega, Norcross, USA).

A total of 300 healthy tilapia individuals were randomly divided into six groups, PBS group, negative control group (shRNA-Control) and four RNAi group (shRNA-199, shRNA-209, shRNA-257 and shRNA-377) with 50 fish per group and reared for 2 weeks. Subsequently, PBS (100 μL), shRNA-Control plasmid (50 μg dissolved in 100 μL PBS) or the four positive shRNA plasmids (50 μg dissolved in 100 μL PBS) was injected into the fish. The liver and spleen were collected at three time points (3, 5 and 7 d) after injection as mentioned above. At each time point, three fish from each group were collected for total RNA extraction as mentioned above. At 12 d after plasmid injection, five fish from each group were collected. The liver and spleen were then collected for morphological and histological examination.

Daily statistical morbidity was calculated for 14 d, and survival rate (SR) was computed by the following iterative calculation formula (SR of 0 day is 1).

$$\text{SR}\;=\;(1\;-\;\frac{Fatality\;fish}{Survival\;fish_{\left(previous\;day\right)}-sampled\;fish})\;\times \;SR_{\left(previous\;day\right)}\;\times \;100\%$$

The knockdown efficiency of the mRNA level was determined *via* qRT-PCR. Moreover, three interaction genes of *lect2*, namely, *peroxisome proliferator activated receptor gamma* (*PPARγ*), *matrix metallopeptidase 2* (*MMP2*) and *β-catenin* (3, 34, 35), were assessed at 5 and 7 d after injection.

### Preparation of On-LECT2 Recombinant Protein (rOn-LECT2)

The specific primers, namely, On-LECT2 recombinant protein-S (rOn-LECT2-S) and rOn-LECT2-A, with the restriction sites *Bam* HI and *Xho* I were designed (**Table S1**) to amplify On-LECT2 ORF without the signal peptide domain. This DNA fragment was purified and ligated with pMD-19T plasmid (TaKaRa, Dalian, China). The recombinant plasmids pMD-19T-rOn-LECT2 and pET-N-His-C-His (Beyotime, Shanghai, China) were digested with *Bam* HI and *Xho* I and then purified. The digested products were then ligated and transformed into *Escherichia coli* BL21 (DE3) (TransGen, Beijing, China). The positive clone was verified *via* PCR and DNA sequencing and then picked to culture in fresh Luria-Bertani (LB) liquid medium containing kanamycin (100 µg/mL) until absorbance at OD600 reached 0.4–0.6. Subsequently, isopropyl-β-d-thiogalactoside (IPTG) was added to a final concentration of 0.5 mmol/L. The bacteria were collected and washed with PBS. The protein was purified with a His-tag protein purification kit (Beyotime, Shanghai, China) and then analysed by 12% reducing SDS-PAGE and Western blot with the His antibody (Beyotime, Shanghai, China).

### Phagocytosis Assay

Monocytes/macrophages were prepared following the method of a previous study (31, 36). 95 µL of *S. agalactiae* with a final concentration of 1×10^5^ CFU/mL was mixed with 95 µL of monocytes/macrophages with an equal final concentration. Afterwards, 10 µL of PBS or 0.5 μg of rOn-LECT2 dissolved in 10 µl of PBS was added. The culture was continued at 28°C, and then 10 µL of the mixture was collected at three time points (2, 4 and 6 h) after the addition. Subsequently, the mixture (10 µL) was continued cultured on a BHI tablet at 28°C for 36 h to count the number of *S. agalactiae*. For each tablet, 10 clones were randomly picked and sequenced the 16s rRNA genes to verify the bacterial species. Each group was repeated in six parallels.

### On-LECT2 Function and Molecular Mechanism Assay

A total of 250 healthy tilapia individuals were randomly divided into five groups with 50 fish per group and reared for 2 weeks. For the blank, PBS and LECT2 groups, 100 μL of PBS was injected into each fish. For the shRNA-Control and shRNA-372 groups, 50 μg of the negative control plasmid pGenesil-1-shRNA-Control dissolved in 100 μL PBS and 50 μg of the most efficient RNAi plasmid pGenesil-1-shRNA-372 dissolved in 100 μL PBS was injected into each fish, respectively. Except for the blank group, the four groups were challenged at 5 d after the first injection with a final concentration of 5×10^7^ CFU/mL *S. agalactiae*. By comparison, the LECT2 group was co-injected with 5 μg of rOn-LECT2 dissolved in the same 100 µl of PBS with *S. agalactiae*. At five time points (0, 6, 12 24 and 48 h) after challenge, the brain, head kidney, liver and spleen tissues from three fish were collected for total RNA extraction as mentioned above and one time point (48 h) for histological analysis. At 48 h after challenge, 10 mg of the liver and spleen tissues of the last four groups was collected, broken and dissolved in 1 mL of PBS and then diluted at a ratio of 1:500 in PBS. Afterward, 50 μL solutions was continued cultured on a BHI tablet at 28°C for 36 h to count the number of *S. agalactiae*. Each group was repeated in six parallels. Daily statistical morbidity was calculated for 7 d from the challenge, and SR was calculated as mentioned above.

*Via* qRT-PCR, the effects, functions and potential molecular mechanisms of On-LECT2 were further investigated by assessing a series of related genes (**Table S1**), including potential receptors, inflammatory factors and typical factors of immune-related pathways.

### Drawings and Statistical Analysis

Drawings and final panels were designed using Adobe Photoshop CC (San Jose, CA, USA) and Adobe Illustrator (San Jose, CA, USA). All data were presented as means ± standard deviation (SD) and the one-way ANOVA and Student’s t test was employed in this study to analyze the significant difference using Prism software (Version 8.0). Different letters or asterisks were marked to illustrate statistically significant differences (*p*<0.05).

## Results

### Characteristics of *On-lect2*

**Figure 1:**
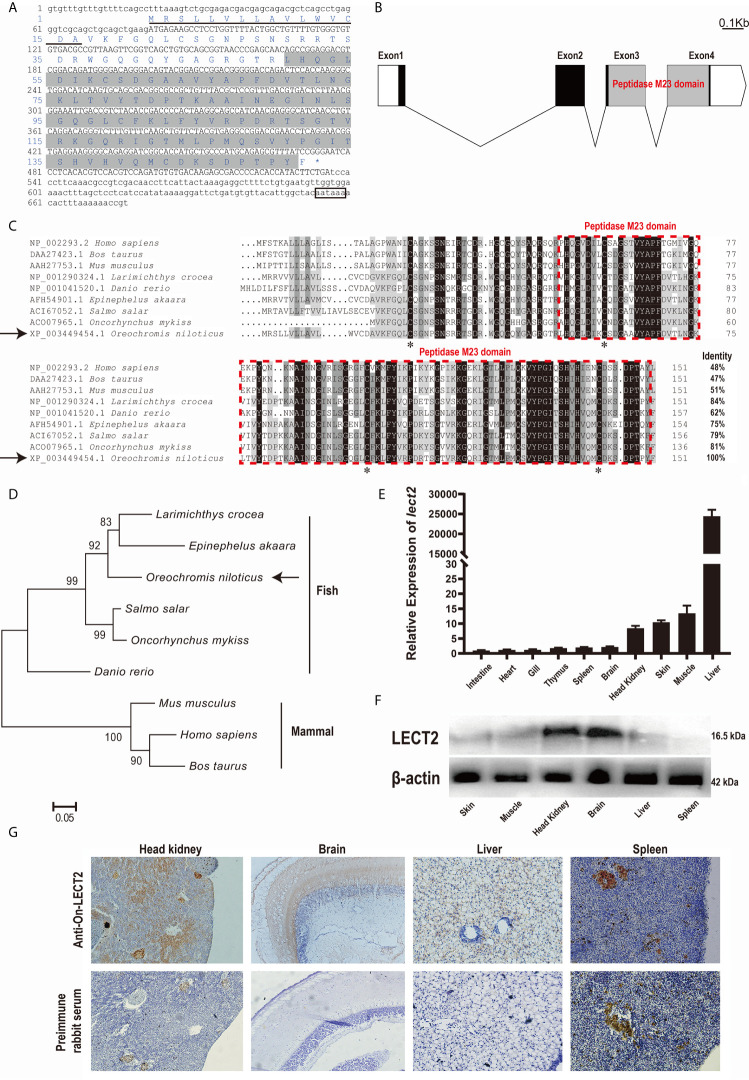
**(A)** cDNA sequences and deduced amino acid sequences of On-LECT2. Open reading frame (ORF) is capitalised, asterisk indicates the stop codon, putative signal peptide is underlined, the peptidase M23 domain is shaded, and putative polyadenylation signal is boxed. **(B)** Genomic structure of *On-lect2*, including four exons and three introns. White box indicates untranslated regions (UTR), blank box denotes ORF and grey box signifies the peptidase M23 domain. **(C)** Multiple sequence alignments of LECT2 from different species. Consensus residues are marked in black. Red dotted box indicates the peptidase M23 domain. Percentage value following each amino acid sequence represents overall sequence identity between On-LECT2 and other sequences. The four conserved cysteine residues are indicated with asterisks below the alignment. **(D)** Phylogenetic tree of On-LECT2 family members constructed using the neighbour-joining method. **(E)** *On-lect2* mRNA levels in different tissues in healthy Nile tilapia as determined by qRT-PCR. All values are the mean ± SD; n=3. Expression level of *On-lect2* in intestine was set as 1. **(F)** Western blot analysis of LECT2 in different tissues of healthy Nile tilapia. **(G)** Localisation of LECT2 in different tissues of healthy Nile tilapia as determined *via* immunohistochemistry (IHC). Positive signals with an antibody LECT2 are marked in brown. The negative control is indicated with preimmune serum. All sections were observed at 400× magnification.

The full-length cDNA of On-lect2 is 726 bp. It consists of a 456 bp ORF that encodes a putative protein of 151 amino acids, including a signal peptide domain of the N-terminal. However, On-LECT2 has no transmembrane domain. The predicted molecular mass of On-LECT2 is 16.5 kDa, and its theoretical p*I* is 9.37. A comparison of genomic sequences revealed that On-lect2 is comprised of four exons that are separated by three introns (**Figures 1A, B**). Multiple sequence alignments indicated that the deduced amino acid sequence of On-LECT2 contains the conservative peptidase M23 domain (**Figures 1A–C**). BLAST analysis revealed that the deduced amino acid sequence is homologous with other LECT2, which is 62%–84% identical to other fish species and about 50% identical to mammals (**Figure 1C**). Phylogenetic analysis suggested that On-LECT2 initially clustered with *Epinephelus akaara* and *Larimichthys crocea* and then clustered with other fish lineages. Finally, On-LECT2 clustered with mammals LECT2 (**Figure 1D**).

### Expression Characteristics of *On-lect2* Among Different Tissues

The expression characteristics of *On-lect2* in different tissues of healthy tilapia at the transcriptional level was measured *via* qRT-PCR (**Figure 1E**). The highest *On-lect2* expression was observed in the liver, whereas it was relatively high in the muscles, skin, and head kidney (**Figure 1E**). The relative expression of On-LECT2 among these tissues were detected *via* Western blot and IHC. On-LECT2 was mainly expressed in the brain and head kidney, followed by in the liver and muscle. The lowest expression level of On-LECT2 was observed in the spleen (**Figures 1F, G**).

### Expression Characteristics of *On-lect2* After Bacteria Challenge

**Figure 2:**
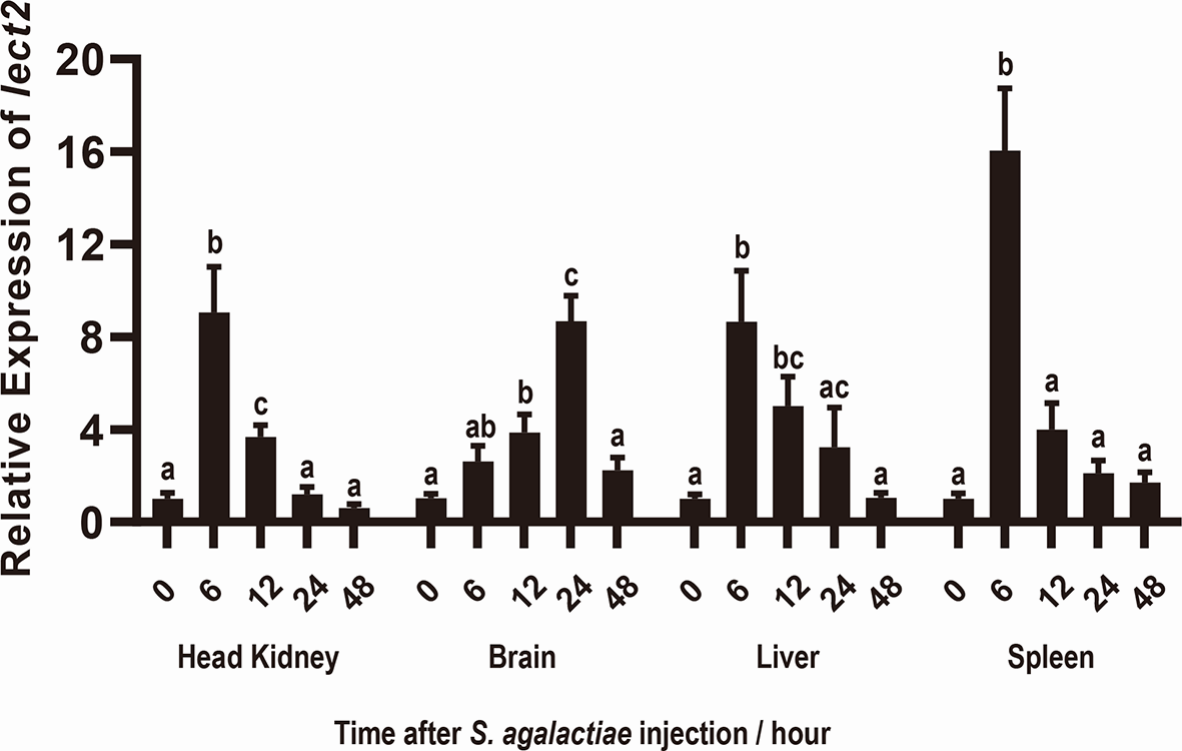
Expression patterns of *On-lect2* in brain, head kidney, liver and spleen tissues at different time points after *S. agalactiae* injection as detected by qRT-PCR. The expression level of *On-lect2* at 0 hour is set as 1.00 to calculate the relative expression of the other time point. All values are the mean ± SD, n=3. Different letters indicated significant difference (*p* < 0.05).

After S. agalactiae infection, the transcriptional levels of On-lect2 significantly increased (*p*<0.05) in the brain, head kidney, liver and spleen tissues. Additionally, the highest transcriptional level of On-lect2 in the head kidney, liver, and spleen was reached at 6 h after the challenge, but it was reached at 24 h in the brain after the challenge (**Figure 2**).

### Silencing of *On-lect2* by Using RNAi

**Figure 3:**
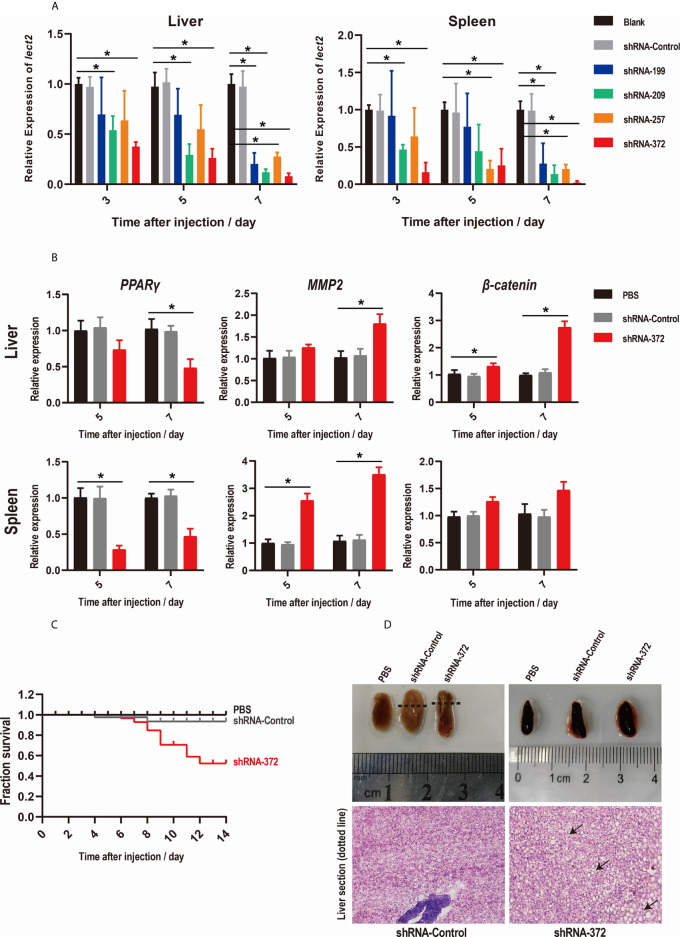
**(A, B)** Levels of *On-lect2* mRNA **(A)**, *PPARγ*, *MMP2* and *β-catenin* mRNA **(B)** in the liver and spleen as detected by qRT-PCR after RNAi plasmid injection. All values are the mean ± SD; n=3. The expression level of *On-lect2* mRNA of the PBS group is set as 1.00 to calculate the relative expression of the other groups. Significant difference (*p*<0.05) in the shRNA-199/209/257/372 groups compared with the Blank or PBS group was indicated by asterisks. **(C)** Survival rates of tilapia after RNAi plasmid injection. Daily statistical morbidity was calculated for 14 days, n=50 for each group. **(D)** Morphological and histological examination of liver and spleen after RNAi plasmid injection. Dotted lines indicate section location. The sections were stained with H&E (400× magnification). Arrows point to vacuolation.

The knockdown efficiency of RNAi plasmids was evaluated by assessing the transcriptional levels of *On-lect2* in the liver and spleen *via* qRT-PCR after RNAi plasmid injection at 3, 5 and 7 d. Unlike in the PBS group, *On-lect2* expression significantly decreased (*p* < 0.05) in all four RNAi plasmid (shRNA-199/209/257/372) injection groups, whereas the control plasmid (shRNA-Control) group was not remarkably different (**Figure 3A**). The silencing efficiency of the RNAi plasmids in the liver was higher than that in the spleen. The levels of *On-lect2* of the shRNA-372 group was the fastest and the largest to decrease (**Figure 3A**). The knockdown efficiency of RNAi plasmids was further evaluated by assessing the transcriptional levels of the three interaction genes (*PPARγ*, *MMP2* and *β-catenin*) of *On-lect2* in the liver and spleen *via* qRT-PCR after shRNA-372 plasmid (the most efficient RNAi plasmid) injection at 5 and 7 d (**Figure 3B**). Compared with that of the PBS group, the transcriptional levels of these genes significantly decreased (*p*<0.05) for *PPARγ* or increased (*p*<0.05) for *MMP2* and *β-catenin* (liver only).

### Functions of *On-lect2* as Revealed by RNAi

SRs were evaluated to explore the functions of On-LECT2 in tilapia at the macroscopic level. Compared with that of the PBS group, the SR of the shRNA-372 group started decreasing from 6 d after plasmid injection and finally decreased below 53% at 12 d, whereas the SR of the shRNA-Control group was still >90% (**Figure 3C**). The liver of the shRNA-372 group showed necrotic foci, haemorrhage and splenomegaly (**Figure 3D**, top lane). The sections stained with H&E showed differences in the features of the liver between the shRNA-Control group and the shRNA-372 group (**Figure 3D**, bottom row). Vacuolation were observed in the liver of the shRNA-372 group (**Figure 3D**, bottom row).

### Effects of rOn-LECT2 on the Promotion of Phagocytosis of Monocytes/Macrophages

**Figure 4:**
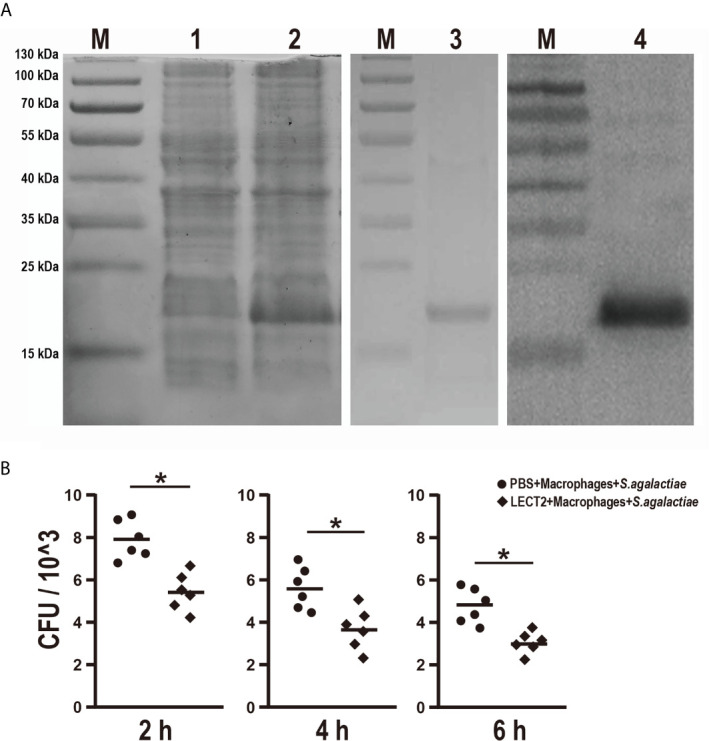
**(A)** SDS-PAGE and Western blot of rOn-LECT2. Lane M, markers; lane 1, bacteria before IPTG induction; lane 2, bacteria after IPTG induction; lane 3, purified rOn-LECT2; lane 4, Western blot analysis of rOn-LECT2. **(B)** Effects of rOn-LECT2 on phagocytosis activity of head kidney-derived macrophages. All values are the mean ± SD; n=6. Significant difference is indicated by asterisks (*p* < 0.05).

The effects of rOn-LECT2 on the promotion of phagocytosis of monocytes/macrophages were determined by constructing the prokaryotic recombinant vector pET-N-His-C-His-On-LECT2 and inducing it to produce the recombinant protein (rOn-LECT2). Subsequently, this recombinant protein (with a predicted molecular weight of 20 KDa) was confirmed *via* SDS-PAGE and Western blot (**Figure 4A**). Furthermore, PBS or rOn-LECT2 was incubated with the premix of macrophages and *S. agalactiae*. Bacterial number was counted at three time points (2, 4 and 6 h). Compared with that of the PBS group, the bacterial number of the rOn-LECT2 group all significantly decreased (*p*<0.05) at three time points (**Figure 4B**).

### Relationship Between On-LECT2 and the Potential Receptors CD209 and CLR

**Figure 5:**
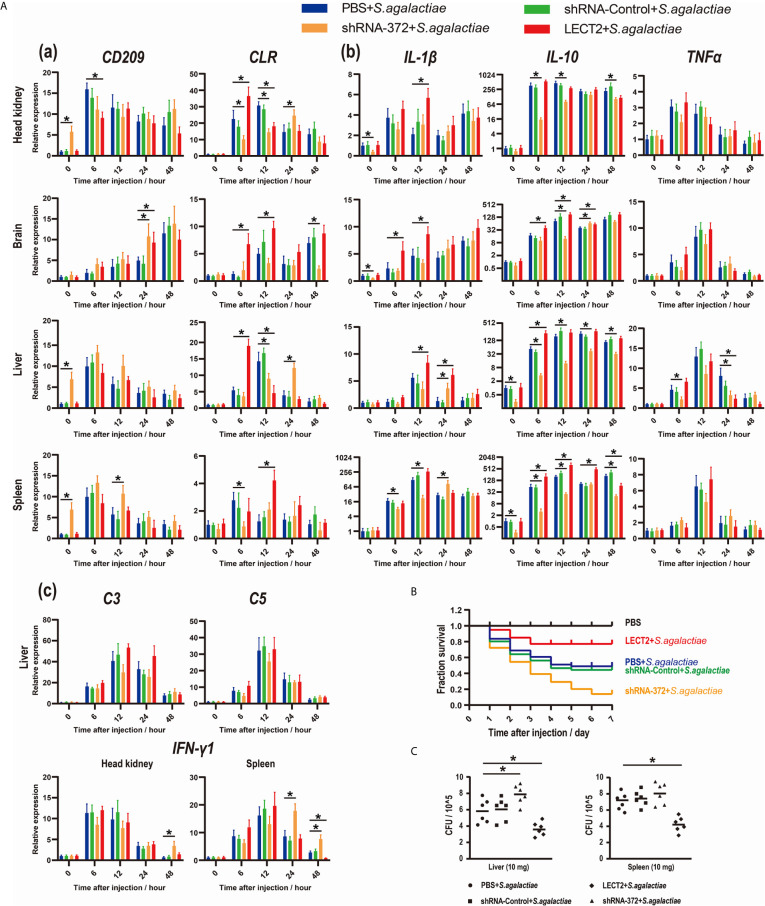
**(A)** Expression patterns of **(a)** potential receptors of LECT2 (*CD209* and *CLR*), **(b)** inflammatory factors (*IL-1β*, *IL-10*, and *TNFα*), **(c)** complements (*C3* and *C5*) and interferon (*IFN-γ1*) as detected by qRT-PCR at different time points after *S. agalactiae* infection. All values are the mean ± SD; n=3. Significant difference between the LECT2 group or the shRNA-372 group and the PBS group is indicated by asterisks. **(B)** Survival rates of Nile tilapia after *S. agalactiae* infection. PBS for blank group, PBS group and LECT2 group, RNAi plasmids for shRNA-Control group and shRNA-372 group in first injection. Five days after the injection, the last four groups (without the blank group) were injected with *S. agalactiae* (5×10^7^ CFU/mL, 100 μL per fish), whereas the LECT2 group was co-injected with 5 μg of rOn-LECT2. Daily statistical morbidity was calculated for 7 days, n=50 for each group. **(C)** Bacterial burden in the liver and spleen 48 h after challenge with *S. agalactiae*. All values are the mean ± SD; n=6. Significant difference is indicated by asterisks (*p*<0.05).

The functions of On-LECT2 in the immune response of Nile tilapia against bacterial infection was further determined by detecting the expression patterns of the potential receptors CD209 and CLR *via* qRT-PCR. The PBS, shRNA-Control, shRNA-372 and LECT2 groups were challenged with *S. agalactiae*. The transcriptional levels of *CD209* and *CLR* in the brain, head kidney, liver and spleen tissues in all groups increased after the bacterial challenge (**Figure 5Aa**). Compared with that of the PBS group, *CLR* expression in the LECT2 group or shRNA-372 group significantly increased (*p*<0.05) or decreased (*p*<0.05) at various time points, respectively (**Figure 5Aa**). However, the difference in *CD209* expression between the PBS and LECT2 groups was not significant. Interestingly, the expression levels of *CD209* in the shRNA-372 group were increased (*p*<0.05) at different time points in the head kidney, liver and spleen tissues (**Figure 5Aa**).

### Effects of On-LECT2 on the Expression of Inflammatory Factors, Complements and Interferon

Inflammatory factors, complements and interferon were also analysed. The transcriptional levels of these genes among the tissues in each group all increased after bacterial infection (**Figures 5Ab** and **5Ac**). Moreover, *IL-1β* and *IL-10* significantly increased (*p*<0.05) or decreased (*p*<0.05) at different time points in the LECT2 group or the shRNA-372 group, respectively (**Figure 5Ab**). However, the expression levels of *TNFα*, *C3* and *C5* were not significantly different among these groups, except for *TNFα* at a few time points in the liver of the shRNA-372 group compared with that of the PBS group (**Figures 5Ab** and **5Ac**). The expression level of *IFN-γ1* in the shRNA-372 group significantly increased (*p*<0.05) at a certain time point in the head kidney and spleen tissues (**Figure 5Ac**).

### Effects of On-LECT2 on the Immune Response of Nile Tilapia Against Bacterial Infection

**Figure 6:**
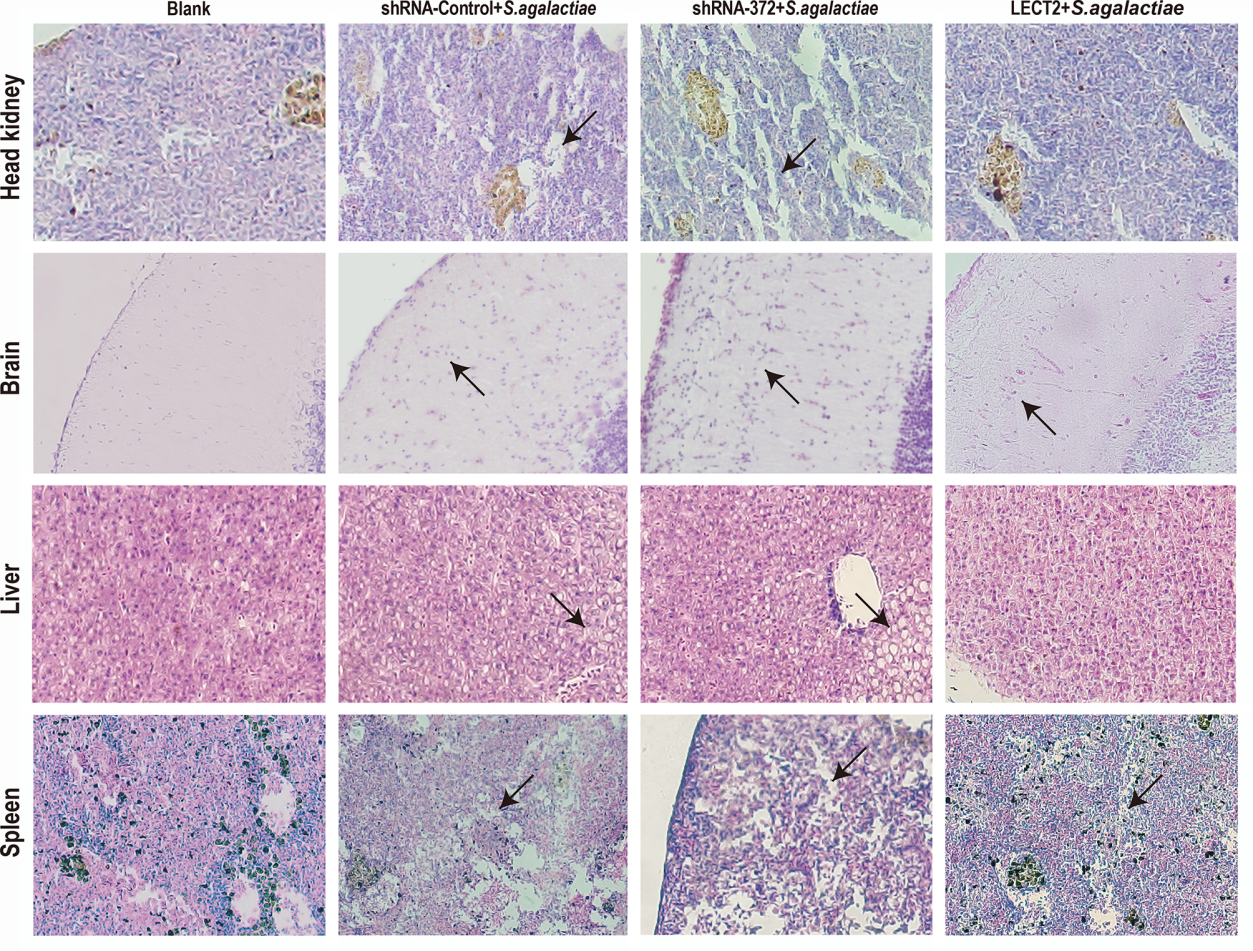
Histology of the head kidney, brain, liver and spleen tissues after *S. agalactiae* infection. Sections were stained with H&E (400× magnification). Arrows point to necrotic foci or haemorrhage.

The SR of the blank, PBS, shRNA-Control, shRNA-372 and LECT2 groups was 100%, 49%, 46%, 13% and 76%, respectively (**Figure 5B**). Furthermore, the bacterial number in the liver of the LECT2 group or the shRNA-372 group significantly decreased (*p*<0.05) or increased (*p*<0.05), respectively, compared with that of the PBS group or the shRNA-Control group, respectively. A similar phenomenon was observed in the spleen (**Figure 5C**). The sections stained with H&E showed different levels of pathological changes. These changes were most obvious in the shRNA-372 group and only mild in the LECT2 group (**Figure 6**).

### Effects of On-LECT2 on Immune-Related Pathways

**Figure 7:**
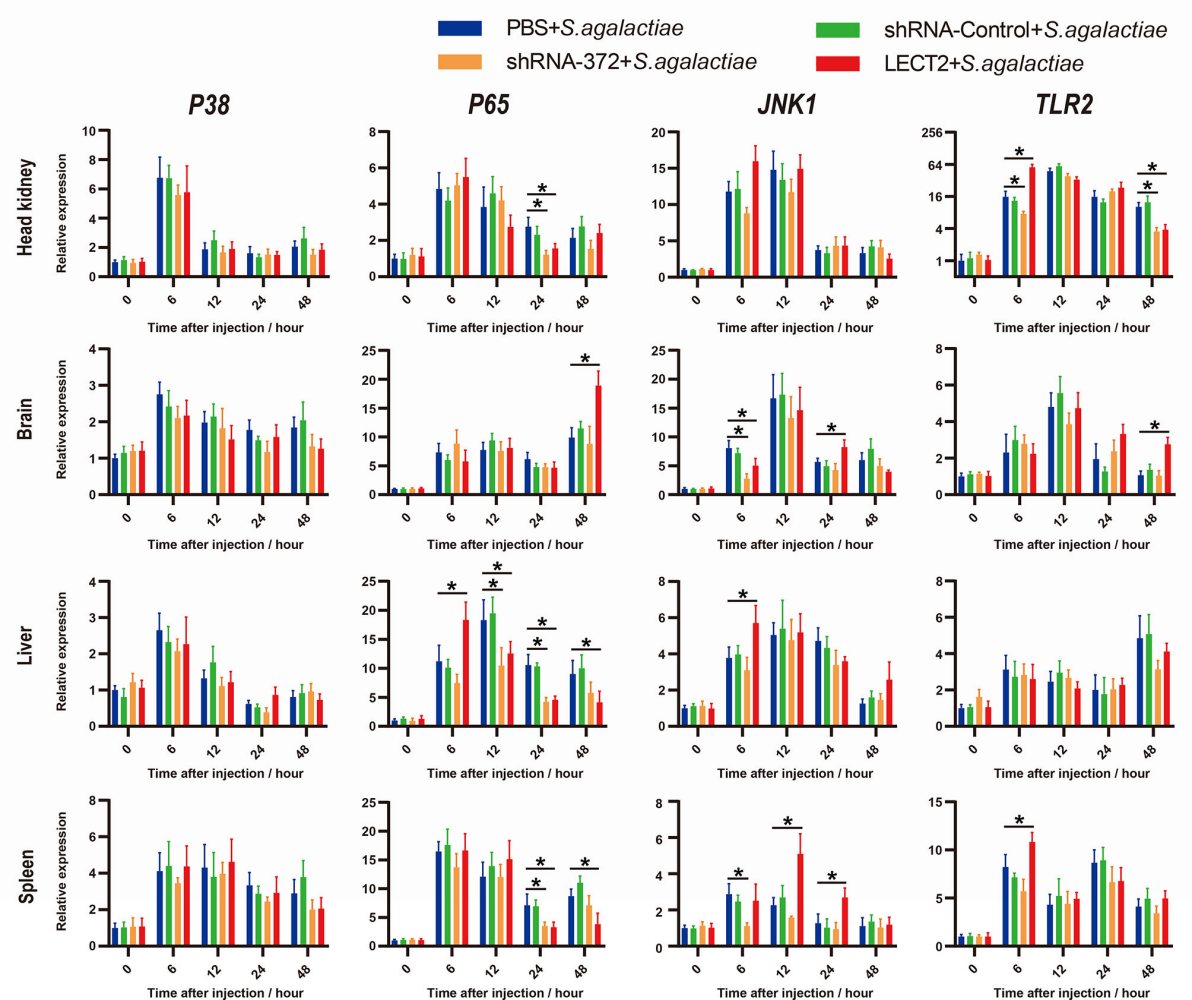
Expression patterns of typical factors of four immune-related pathways as detected by qRT-PCR at different time points after *S. agalactiae* infection. All values are the mean ± SD; n=3. Significant difference between the LECT2 group or the shRNA-372 group and the PBS group is indicated by asterisks.

Similarly, the expression levels of four typical immunological factors, namely, *P38*, *P65*, *JNK1* and *TLR2*, each of which represents an immune-related pathway, all increased after bacterial infection (**Figure 7**). The transcriptional levels of *P65*, *JNK1* and *TLR2* in the LECT2 group or the shRNA-372 group significantly increased (*p*<0.05) or decreased (*p*<0.05), respectively, compared with that of the PBS group (**Figure 7**). Remarkably, the difference in the transcriptional level of *P38* among these groups was not significant (**Figure 7**).

## Discussion

In this study, we identified and characterised LECT2 from Nile tilapia. The deduced On-LECT2 is a secreted protein that contains a conserved peptidase M23 domain and shares over 60% similarity with other fish LECT2 and about 50% similarity with mammal LECT2. This result highlighted the evolutionary importance of LECT2 because it is highly conserved across a wide variety of species. Moreover, three disulphide bonds exist between six evolutionarily conserved cysteine residues in mammals, cyprinids and catfish (1, 23, 37). However, consistent with most teleost species (19, 22, 37), On-LECT2 contains four conserved cysteine residues and lacks the second and third cysteine residues, implying that LECT2 might had been passed down through several generations and modified in most fish lineages.

Similar to higher vertebrates and other fish lineages (18, 23, 24, 38), the highest transcriptional level of *On-lect2* was detected in the liver, whereas the lower levels were observed in the other tissues. Furthermore, the transcriptional levels of *On-lect2* in the muscle, skin and head kidney were relatively high, similar to that observed in groupers (39) and asian seabass (38). This result implied that On-LECT2 is possibly involved in the immune response against pathogens (36). Interestingly, the expression level of *lect2* is also relatively high in the spleen in other fish species, such as rainbow trout (19), croceine croaker (32) and grass carp (23). This observation is quite understandable because the spleen is one of the most important peripheral lymphoid organs in fish (40). In humans, LECT2 is generally detected in vascular, endothelial and smooth muscle cells, cerebral nerve cells, and some mononuclear cells in immune hematopoietic tissues (2). In this study, On-LECT2 protein was mainly detected in the brain and head kidney, confirming that most of the On-LECT2 protein is immediately secreted after synthesised.

The mechanism by which On-LECT2 functions in the immune response against bacterial infection was clarified by investigating its transcriptional levels in different tissues *via* qRT-PCR. Results showed that *On-lect2* expression significantly increased after *S. agalactiae* infection. In addition, the peak *On-lect2* expression was reached at 6 h in the head kidney, liver, and spleen, but it was reached at 24 h in the brain because the blood–brain barrier likely resisted the penetration of bacteria (31, 41). The highest and most rapid increase in *On-lect2* expression was detected in the spleen, one of the major immune organs in teleosts that contains various immune cell types, including macrophages, B cells and T cells (40).

Notably, 4% PFA, one of the most common fixatives used for histological examination, was not effective for fixing tilapia liver and spleen tissues. This observation was reported by Ellis in zebrafish (42). Unfortunately, our initial samples were damaged after fixation with 4% PFA. Some portions of the liver and spleen tissues fell off of the sections, the liver and spleen tissues shrunk and the cytoplasm of the hepatocytes was almost completely lost (**Figure S1**), similar to that observed in zebrafish (42). Thus, we had to repeat the experiment and collect additional samples for subsequent experiments.

In this study, RNAi was applied for the first time to investigate the functions of immune genes in tilapia. RNAi plasmids were injected into the fish similar to that conducted in zebrafish (43, 44). Results showed that the expression level of *On-lect2* in the liver and spleen significantly decreased (*p*<0.05). Moreover, the three interaction genes of *On-lect2* either significantly decreased or increased. Furthermore, we evaluated the expression levels of LECT2 after *S. agalactiae* infection between control group and RNAi group *via* IHC (**Figure S2**), the positive signals of LECT2 were less in RNAi group. Besides, higher mortality was observed in the RNAi group due to liver vacuolation, necrosis and splenomegaly, suggesting that LECT2 is indispensable for the maintenance of liver and spleen functions in tilapia. In addition, the expression of LECT2 is often decreased during tissue inflammation, fibrosis and pathology in humans and mice. However, in fish species, including tilapia, the expression of *lect2* increased after bacterial infection (22, 23). The exact functions of On-LECT2 were further investigated by preparing the recombinant protein for *in vitro* study. Similar to that observed in mice, ayu and lamprey (7, 22, 45), On-LECT2 substantially improved the phagocytic activity of head kidney-derived macrophages. These data suggested the importance of LECT2 in the regulation of liver functions or immune responses in fish and mammals.

*In vivo* challenge experiments were conducted to systematically analyse the relationships between On-LECT2 and immune-related genes or pathways. Results suggested CLR rather than CD209 was the most likely receptor of On-LECT2 because only *CLR* was markedly decreased or increased in the shRNA-372 group or the LECT2 group, respectively. These results were consistent with those reported in ayu (46, 47), implying that this different bonding form is likely conserved in fish. Our data showed that On-LECT2 improved the SR and reduced bacterial tissue damage after *S. agalactiae* infection (**Figures 5B, C** and **6**). Similar conclusions were also drawn from mice and fish (7, 48). Interestingly, qRT-PCR detection results of inflammatory factors, complements and interferon during bacterial challenge suggested that only the inflammatory factors *IL-1β* and *IL-10* (except for *TNFα*) were regulated by On-LECT2, consistent with the results detected by qRT-PCR in mice macrophages after *E. coli* injection. ELISA also demonstrated that IL-1β, IL-10, C3 and IFN-γ are regulated by LECT2 after LPS stimulation in mice (7). Another study reported that only the serum levels of TNFα and IFN-γ and the transcriptional level of *IFN-γ* in liver are substantially changed in LECT2-deficient mice after LPS/D-GalN injection (49). Similar studies in ayu indicated that the transcriptional levels of *IL-1β*, *IL-10* and *TNFα* were down regulated by LECT2 after *Vibrio anguillarum* infection (48). These results might be attributed to the different mechanisms of immune responses in mammals and fishes, as well as the different bacteria used in the challenge (*S. agalactiae* is Gram-positive, whereas *E. coli* and *V. anguillarum* are Gram-negative).

In humans and mice, the expression of *P38*, *P65* and *JNK1* as regulated by LECT2 were confirmed. LECT2 reportedly promotes inflammation and bactericidal ability *via* the CD209a receptor (50–53). The relationship between LECT2 and Toll-like receptor signalling was reported in chicken. *chlect2* might be a target gene of the TLR3 signalling (54). *P65* and *JNK1* were also identified from LECT2-influenced genes of ayu macrophages (8). The relationship between *P38* or *TLR2* and LECT2 has not been established in fish. Our data indicated that *P65*, *JNK1* and *TLR2* might be regulated by On-LECT2, whereas *P38* appears to be not involved (**Figure 7**). LECT2 stimulates inflammation *via* the CD209/P38-dependent pathway in the adipocytes of human liver (50). Instead of CD209, CLR, while working as the LECT2 receptor, might induce different downstream pathways in fish. Notably, *TLR2* was considerably upregulated by On-LECT2 during bacterial challenge, especially in the head kidney, one of the most important immune organs in teleost (40). As a member of the Toll-like receptor (TLR) family, TLR2 recognises pathogen-associated molecular patterns and induces the production of cytokines in fish (55). Additional functional studies are warranted to obtain a better understanding of the interaction between LECT2 and TLR2 and to clarify the roles of LECT2 in the regulation of the immune response to bacterial infection in teleosts.

In summary, our study identified a fish leukocyte cell-derived chemotaxin 2 homolog (i.e., On-LECT2) and determined its involvement in the immune response of Nile tilapia against bacterial infection. On-LECT2 was mainly synthesised in the liver and functions by promoting inflammation, reducing tissue damage and improving SR. This study lays a theoretical foundation for further investigation into the mechanism by which LECT2 protects fish against pathogens.

## Data Availability Statement

The original contributions presented in the study are included in the article/**Supplementary Material**. Further inquiries can be directed to the corresponding authors.

## Ethics Statement

The animal study was reviewed and approved by Guangdong Province Laboratory Animal Management Regulations.

## Author Contributions

QL and YH designed the experiments. QL, ZZ, WF, and YXH performed experiments. JN, XL, and GL performed Nile tilapia feeding and cell isolation. QL analyzed the data and wrote the manuscript. JJ and YH reviewed the manuscript. All authors contributed to the article and approved the submitted version.

## Funding

This work was supported by the National Key R&D Program of China (No. 2018YFD0900501), the National Natural Science Foundation of China (No. U20A2065, 32073006, 32002426), the Fund of Southern Marine Science and Engineering Guangdong Laboratory (Zhanjiang) (No. ZJW-2019-06).

## Conflict of Interest

The authors declare that the research was conducted in the absence of any commercial or financial relationships that could be construed as a potential conflict of interest.
